# Identification of a Functional, CRM-1-Dependent Nuclear Export Signal in Hepatitis C Virus Core Protein

**DOI:** 10.1371/journal.pone.0025854

**Published:** 2011-10-24

**Authors:** Andrea Cerutti, Patrick Maillard, Rosalba Minisini, Pierre-Olivier Vidalain, Farzin Roohvand, Eve-Isabelle Pecheur, Mario Pirisi, Agata Budkowska

**Affiliations:** 1 Unité Hépacivirus et Immunité Innée, Département de Virologie, Institut Pasteur, Paris, France; 2 CNRS, URA3015, Paris, France; 3 Dipartimento di Medicina Clinica e Sperimentale, Università del Piemonte Orientale “Amedeo Avogadro”, Novara, Italy; 4 Unité de Génomique Virale et Vaccination, Département de Virologie, Institut Pasteur, Paris, France; 5 Hepatitis and AIDS Department, Pasteur Institute of Iran, Teheran, Iran; 6 Université Lyon 1, Lyon, France; 7 CNRS, UMR5086, Lyon, France; 8 IBCP, Bases Moléculaires et Structurales des Systèmes Infectieux, Lyon, France; St. Petersburg Pasteur Institute, Russian Federation

## Abstract

Hepatitis C virus (HCV) infection is a major cause of chronic liver disease worldwide. HCV core protein is involved in nucleocapsid formation, but it also interacts with multiple cytoplasmic and nuclear molecules and plays a crucial role in the development of liver disease and hepatocarcinogenesis. The core protein is found mostly in the cytoplasm during HCV infection, but also in the nucleus in patients with hepatocarcinoma and in core-transgenic mice. HCV core contains nuclear localization signals (NLS), but no nuclear export signal (NES) has yet been identified.

We show here that the aa(109–133) region directs the translocation of core from the nucleus to the cytoplasm by the CRM-1-mediated nuclear export pathway. Mutagenesis of the three hydrophobic residues (L119, I123 and L126) in the identified NES or in the sequence encoding the mature core aa(1–173) significantly enhanced the nuclear localisation of the corresponding proteins in transfected Huh7 cells. Both the NES and the adjacent hydrophobic sequence in domain II of core were required to maintain the core protein or its fragments in the cytoplasmic compartment. Electron microscopy studies of the JFH1 replication model demonstrated that core was translocated into the nucleus a few minutes after the virus entered the cell. The blockade of nucleocytoplasmic export by leptomycin B treatment early in infection led to the detection of core protein in the nucleus by confocal microscopy and coincided with a decrease in virus replication.

Our data suggest that the functional NLS and NES direct HCV core protein shuttling between the cytoplasmic and nuclear compartments, with at least some core protein transported to the nucleus. These new properties of HCV core may be essential for virus multiplication and interaction with nuclear molecules, influence cell signaling and the pathogenesis of HCV infection.

## Introduction

Hepatitis C virus (HCV) infection is a major cause of chronic liver disease worldwide. Most infected subjects develop a chronic infection that may progress to steatosis, liver cirrhosis and HCC. Current treatment is based on the combination of pegylated interferon alpha and ribavirin, and leads to elimination of the virus in 50 to 80% of cases, depending on the genotype {Shepard, 2005 #1;Tellinghuisen, 2002 #77}. The development of more effective treatments will require improvements in our understanding of the interactions between the virus and host-cell components.

HCV belongs to the *Hepacivirus* genus, within the Flaviviridae family. The HCV genome, a single-stranded RNA of positive polarity, consists of 9,600 nucleotides and encodes a single polyprotein that is cleaved into structural and nonstructural proteins by cellular and viral proteases. Core and the envelope E1 and E2 glycoproteins form the putative viral particle, together with lipoproteins. The nonstructural proteins are involved in the synthesis of HCV RNA and virus assembly.

HCV core protein is cleaved from the polyprotein by cellular proteases (see for review. Signal peptidase cleaves a C-terminal signal sequence between core and the E1 glycoprotein, thereby producing the 191 amino-acid (aa) immature form of core. This form (MW 23 kDa) remains anchored to the endoplasmic reticulum (ER). It is then cleaved by a signal peptide peptidase, which removes the signal peptide to generate the mature form of core (MW. 19–21 kDa), which is 173–179 aa long and is trafficked from the ER membrane to lipid droplets (LDs). The association of the mature core protein with LDs is directly related to the intracellular transport of this protein to the perinuclear area, the site of assembly of infectious HCV particles. HCV is then secreted through the VLDL-secretory pathway.

**Figure 1:**
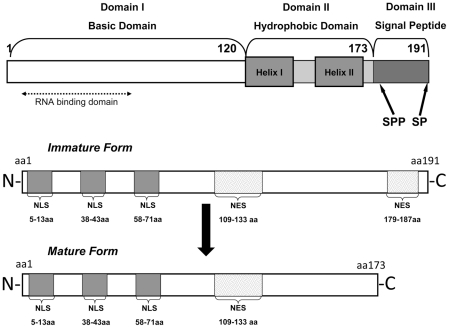
Schematic diagram of structural and functional domains within the HCV core protein. The RNA-binding region aa(1–57), the three nuclear localization signals (NLS), and the classical NES aa(179–187) and the candidate “non classical” NES aa(109–133) identified in this study are shown. Numbers identify the aa positions covered by each domain and functional region.

The core protein has three functional domains: the highly basic N-terminal domain I (DI) is involved in the interaction with HCV RNA; the hydrophobic domain II (DII) contains structural determinants mediating the binding of core to cellular membranes and lipid droplets and domain III (DIII) is a signal peptide that is cleaved during the formation of the mature core protein (Figure 1). When the entire polyprotein is synthesized in mammalian cells, core is found mostly at the ER membrane and on the surface of lipid droplets and mitochondria. HCV core may also be found in the nucleus, where it may act as a substrate for proteasomal degradation, particularly when C-terminally truncated forms of core are produced. These findings suggest that core is targeted away from the ER very soon after its synthesis. However, it remains unclear what determines the ultimate fate of core, whether it remains at the ER or is trafficked to other subcellullar compartments, and the regulation of this process appears to be complex (see for review,).

In addition to binding to the HCV RNA to form the virus nucleocapsid, core protein interacts with several cellular components, thereby influencing lipid metabolism, signal transmission, and the regulation of gene expression and transcription from several viral and cellular promoters. HCV core affects cell proliferation, apoptosis and host defense mechanisms, by interfering with both innate and adaptive immunity (see for review:). Furthermore, studies in transgenic mice and observations in humans suggest that core protein has oncogenic potential. HCV core consistently interacts with several host factors, including the p53 tumor suppressor protein and p21, and LZIP, and with cellular shuttling proteins, such as p53, 14-3-3, DDX3, putative RNA helicase and proteasome activator PA28γ. These particular properties of core may be related to HCV-induced cell transformation (for review, see).

In liver biopsy specimens from HCV-infected individuals, HCV core is found mostly in the cytoplasmic compartment, but a nuclear localization of core has also been reported. In particular, tumor tissues from patients with HCC have been found to contain truncated forms of the HCV core protein within the nucleus. These studies suggested that core may localize to the nucleus at some stages of natural HCV infection, particularly in cancer patients. Similarly, in transgenic mice producing HCV core protein, a nuclear localization of core was associated with liver disease and HCC. The interaction of core protein with nuclear molecules may therefore play a key role in the development of HCC.

Some of the proteins of positive-strand RNA viruses, which replicate in the cytoplasm, may be located in the nucleus or the nucleolus at some point in the viral life cycle. Such proteins contain appropriate targeting signals, such as nuclear localization signals (NLS) and nuclear export signals (NES). The distribution of core protein may thus also be regulated by these signals. Indeed, three NLS have been identified in HCV core, in the aa(5–13), aa(38–43), and aa(58–71) sequences. These sequences constitute functional, at least bipartite NLS able to bind importin-α. However, no NES that could potentially direct the translocation of the protein from the nucleus to the cytoplasm has yet been reported in HCV core.

We demonstrate for the first time that core protein contains a functional NES (aa(109–133)) facilitating its export from the cell nucleus via the CRM-1/exportin pathway. In the HCV *in vitro* replication system (JFH1), HCV core was translocated to the nucleus early in infection. The presence of functional NLS and NES motifs raises the possibility of core protein shuttling between the nuclear and cytoplasmic compartments. These new properties of core may be important for virus multiplication and the pathogenesis of infection.

## Materials and Methods

### Cell culture

All cell lines were cultured at 37°C, in a humidified atmosphere containing 5% CO_2_. The human hepatoma cell line Huh7, the rat hepatoma cell line ARL-6 and human embryonic kidney 293T cells were obtained from ATTC, immortalized human hepatocytes Fa2N-4 were purchased from XenoTech, Lenexa KS, USA, whereas the Huh 7.5 cell line was kindly provided by C. Rice. Cells were maintained in Dulbecco's modified Eagle's medium (DMEM; Gibco-Invitrogen) supplemented with 10% fetal calf serum, 1% non-essential aminoacids and antibiotics (penicillin 100 U/ml and streptomycin 100 µg/ml). The CHO-K1 cell line (ATCC#CCL-61) was obtained from LGC Prochem (Molsheim, France) and cultured in F12 medium (Invitrogen, Cergy-Pontoise, France) containing glutamine and supplemented with 10% fetal calf serum (FCS) antibiotics and anti-fungal compounds.

### Plasmid construction

Plasmids were constructed by inserting core-encoding fragments of various lengths — aa(1–120), aa(1–140), aa(1–160), aa(1–173) — into pEGFPN1 (Clontech). These fragments were amplified by standard PCR, cleaved with *Eco*RI and *Bam*HI and inserted into pEGFPN1.

We constructed several plasmids encoding enhanced green fluorescent protein (EGFP) or mCherry, to determine whether the NES and NLS sequences were functional. The reporter genes were fused with (i) the NLS of SV40 nuclear T antigen (EGFP-NLSSV40), (ii) the NLS of SV40 followed by the potential NES of the HCV core protein aa(109–133) (EGFP-NLSSV40-NEScore), or (iii) the NLS of SV40 followed by the functional NES of the HIV regulatory protein Rev (EGFP-NLSSV40-NESRev). Three more plasmids, encoding core fragments aa(109–160), aa(144–160) and aa(134–143), were constructed. These fragments were fused with the EGFP or mCherry sequence and the NLS of SV40 (EGFP-NLSSV40-core(109–160); EGFP-NLSSV40-core(144–160); EGFP-NLSSV40-core(134–143)).

All viral coding sequences were amplified by standard PCR and introduced into the pDONR207 plasmid (Invitrogen) via a recombinational cloning strategy (Gateway; Invitrogen). Viral sequences were then transferred from pDONR207 into Gateway-compatible versions of pEGFP-C1 (kindly provided by Y. Jacob) or mCherry vectors (Clontech), for expression in mammalian cells with an N-terminal EGFP or mCherry tag.

The PCR primers used to amplify and clone viral ORFs had 20 to 30 specific nucleotides matching the ends of the ORF, giving a Tm close to 60°C. For the recombinational cloning of PCR products, the 5′ ends of forward primers were fused to the attB1 5′-GGGGACAAGTTTGTACAAAAAAGCAGGCATG-3′ recombination sequence, whereas the reverse primers were fused to the attB2 5′-GGGGACCACTTTGTACAAGAAAGCTGGTTA-3′ recombination sequence. All the constructs used were amplified by transforming (by heat shock) *Escherichia coli* DH5α (Invitrogen).

### Cell transfection

For overexpression, mammalian cells were transfected with pEGFP-C1 or mCherry vectors containing the viral ORFs, in the presence of the FuGene Transfection Reagent (Promega), according to the manufacturer's instructions. Unless otherwise specified, we dispensed 5x10^4^ cells per into each well of 24-well plates and, 24 to 48 h later, we transfected these cells with 500 ng of plasmid DNA per well.

### Mutations in the NES sequence

Mutations (L119A, I123A, and L126A) were introduced into the NES sequence of HCV core protein with the QuikChange Lightning site-directed mutagenesis kit (Agilent-Stratagene). An EGFP expression plasmid (EGFP-NLSSV40-NEScore) encoding the putative HCV core export sequence aa(109–133) or EGFP-labeled core protein aa(1–173) was amplified with the 5′-phosphorylated mutagenic primers 5′-GCCGATACCGCTACATGCGGCTTCGCCGACCTCAT and 5′-GACCTTACCCGCATTACGCGACCTACGCCGGGGGT. Template DNA was then digested with *Dpn*1 and mutagenized plasmids were circularized by self-ligation with T4 DNA Ligase (New-England Biolabs). Mutant clones were selected and the sequences of their expression cassettes were checked.

### Sequence analyses

Multiple sequence alignment was carried out with Clustal W. “Classical” NES were identified with NetNes software (www.expasy.org).

### HCVcc (JFH1) cell culture

The plasmid corresponding to the genome of the JFH1 strain was kindly provided by T. Wakita and used to generate cell culture-produced virus (HCVcc). The virus was cultured as previously described. For cell infection, monolayers of Huh 7.5 cells were grown for 24 h in tissue culture plates. They were then inoculated by incubation with 25 µl of the virus preparation (containing approximately 10^6^ IU of HCV RNA) for 2 h at 37°C, to allow infection. Cells were analyzed at various time points after infection, as indicated, by immunofluorescence studies, quantitative RT-PCR or electron microscopy.

### RNA interference

For RNA interference-based knockdown experiments, a 25-nucleotide siRNA (ccu cgu ugc uga agg ugg auc agg a) targeting PA28γ was purchased from Invitrogen (Stealth Select RNAi). Huh7.5 cells were tranfected with 20 nM siRNA, in the presence of JetPRIME (Ozyme). Control ON-TARGET plus (Dharmacon) non targeting siRNA was used to confirm silencing specificity. For analyses of the influence of proteasome activator silencing on the subcellular distribution of core, cells were transfected with siRNA targeting PA28γ or with control siRNA 18 h before infection with HCVcc.

### LMB treatment

We investigated the role of CRM-1-dependent transport in the subcellular localization of core, by treating cells with leptomycin B (LMB, Sigma). Huh7 cells were cultured and transfected as described above. After 22 to 48 h, the cells were treated for 2 to 4 h, at 37°C, with LMB at a concentration of 2 ng/ml or 10 ng/ml in DMEM. For studies in the JFH1 infection model, 10 ng/ml LMB was applied at various time points during or after infection, and cells were incubated in a medium containing LMB for the indicated times.

Cell viability after LMB treatment was determined by counting live and dead cells, after trypan blue staining, in an automated cell counter (Countess; Invitrogen). We also quantified the ATP present in cultured cells, with the CellTiter-Glo® Assay (Promega), used according to the manufacturer's instructions. Untreated cells and cells treated with 10% DMSO (to induce cell death) were used as negative and positive controls, respectively.

### Immunofluorescence and confocal microscopy

We investigated PA28γ production in untransfected Huh 7.5 cells and in Huh 7.5 cells transfected with an siRNA targeting PA28γ (or control siRNA), with a rabbit anti-PA28γ antibody and Alexa Fluor 488-conjugated anti-rabbit IgG. The distribution of HCV core in infected cells was analyzed with a monoclonal anti-core antibody, ACAP27, followed by an Alexa Fluor 568-tagged anti-mouse IgG. Staining with rabbit anti-lamin B antibody followed by Alexa Fluor 488-conjugated anti-rabbit IgG was used to outline the cell nuclei.

For fluorescence microscopy, infected or transfected cells were washed in PBS and fixed by incubation with 4% paraformaldehyde (PFA) in PBS for 20 min at 4°C. The cells were washed several times in PBS and permeabilized with 0.5% Triton X-100. They were then incubated in a blocking buffer containing 1% gelatin and 0.1% Tween 20 in PBS. Primary antibodies were incubated with the cells for 2 h (mostly at a concentration of 1 µg/ml in blocking buffer). After subsequent washes with 0.1% Tween 20 in PBS, secondary antibodies were added and the cells were incubated for 1 h. The cells were washed again and mounted in Vectashield medium containing DAPI (Vector Laboratories, Abcys, France). Fluorescent fields were captured with a Widefield ApoTome AxioCam upright (66RC10P) microscope.

### Relative fluorescence analyses

We analyzed the relative fluorescence intensity of the proteins in the cytoplasm and nucleus, by converting bright-field immunofluorescence images to grayscale images, with Image J software. Boundaries were applied to demarcate the nuclear and cytoplasmic compartments and the fluorescence intensity of each compartment was measured with a script created with Acapella 2.0 image software (Perkin Elmer). All measurements were normalized with respect to background fluorescence. A nonparametric one-way ANOVA assay was carried out to compare fluorescence between the cytoplasm and the nucleus of 1138, 712 and 573 cells transfected with plasmids (EGFP-NLSSV40), (EGFP-NLSSV40-NESRev) and (EGFP-NLSSV40-NEScore), respectively. Graphs showing the means and variances of the ratio of nuclear to cytoplasmic fluorescence intensities were plotted for the three constructs considered.

For analyses of the mutated NES, 153 cells transfected with the wild-type (EGFP-NLSSV40-NEScore) plasmid and 130 cells transfected with the mutated construct were analyzed, and the results are presented as a graph showing the means and variances of the ratio of nuclear to cytoplasmic fluorescence intensities for wild-type and mutated constructs. Similarly, 139 and 101 cells transfected with the wild-type and mutated core protein aa (1–173) constructs, respectively, were also considered. Graphs showing the means and variances of the ratio of nuclear to cytoplasmic fluorescence intensities were plotted for the wild-type and mutant proteins and a nonparametric *t*-test was used to evaluate the results obtained.

### RT-quantitative PCR (RT-qPCR)

The HCV RNA associated with cells was quantified by one-step real-time RT-qPCR, with the SuperScript III Platinum One-Step qRT-PCR Kit (Invitrogen). The 5′-AGYGTTGGGTYGCGAAAG-3′ and 5′-CACTCGCAAGCRCCCT-3′ primers were used to amplify HCV RNA from JFH clones, and 6-FAM-CCTTGTGGTACTGCCTGA-MGB (Applied Biosystems, Foster City, CA, USA) was used as an internal probe. Real-time detection of the PCR products was carried out with an AbiPrism 7000 machine. HCV RNA was quantified and standardized with an HCV RNA quantification panel from AcroMetrix, and the values obtained are expressed as HCV RNA IU.

### Western Blot

Huh7 cells (1.5×10^4^) were transfected with plasmids encoding core proteins of various lengths — aa(1–140), aa(1–160), aa(1–173) — fused to EGFP. Transfection was performed with jetPRIME™ Transfection Reagent (Polyplus Transfection Company, France), according to the manufacturer's instructions. After 24 h, the transfected cells were washed with PBS and lysed by incubation for 5 min on ice with 900 µl/well 1X RIPA buffer (20 mM Tris-HCl pH7.5, 150 mM NaCl, 1 mM Na_2_EDTA, 1 mM EGTA, 1% NP-40, 1% sodium deoxycholate, 2.5 mM sodium pyrophosphate, 1 mM Na_3_VO_4_, 1 µg/ml leupeptin). Cells were scraped of the plates and collected by centrifugation for 10 min. at 14,000 rpm at 4°C. Supernatants were collected and separated by SDS-PAGE in 10% polyacrylamide gels and the protein bands were transferred to nitrocellulose membranes (Pure Nitrocellulose membrane, Bio-Rad). Proteins were detected by incubation with polyclonal anti-GFP (Roche) and anti-tubulin (Abcam) antibodies, followed by peroxidase-conjugated anti-rabbit immunoglobulin antibodies (Santa Cruz Biotechnology). The proteins bands were detected by enhanced chemiluminescence (Sigma).

### Electron microscopy

Supernatants collected from Huh7.5 cells producing JFH1 were concentrated by centrifugation through a 20% sucrose cushion for 4 h at 32,000 rpm, in an SW32Ti rotor. For electron microscopy analyses, Huh7.5 cells were grown in 3 cm dishes for 24 h, washed with cold serum-free-DMEM and maintained for 10 min at 4°C. The medium was then replaced with 1 ml cold serum-free DMEM supplemented with a concentrated virus preparation (approx. 5×10^9^ IU/ml HCV RNA) and the cells were incubated for 30 min at 4°C. At the end of this adsorption step, the cells were placed for 20 min at 37°C and examined by transmission electron microscopy (TEM). Briefly, cells were fixed by incubation with 4% (v/v) paraformaldehyde (Delta Microscopies, Ayguevive, France) and 0.05% (v/v) glutaraldehyde in phosphate-buffered saline (PBS) pH 7.4, for 30 min at 37°C. They were washed with PBS and incubated for 10 min in blocking solution containing 0.5% v/v cold fish skin gelatin (FSG), 0.1% v/v saponin and 0.02 M glycine (all from Sigma Aldrich) in PBS pH 7.4, at room temperature. The cells were washed with 0.5% FSG in PBS (PBS/FSG) and incubated for 60 min with ACAP-27 monoclonal antibody, kindly provided by JF. Delagneau, (0.6 µg/ml, diluted in PBS/FSG). The cells were again washed in PBS/FSG, incubated for 30 min with anti-mouse IgG conjugated with Nanogold®particles (Yaphank, NY, USA) in PBS/FSG, washed in PBS/FSG and fixed by incubation with 1% v/v glutaraldehyde in PBS for 1 h at room temperature. The cells were washed in water and Nanogold® staining was enhanced by incubation for three minutes with the HQ silver enhancement kit (Nanoprobe, Yaphank, NY, USA). Cells were then post-fixed by incubation with 1% v/v osmium tetroxide in PBS for 1 h and processed by dehydration in a series of ethanol solutions for embedding in epoxy resin. TEM examination was performed on a Philips CM120 microscope, and images were acquired with an ORIUS SC200D CCD camera (Gatan Inc.).

## Results

### Subcellular localization of core proteins

Several studies have shown that, when produced separately, the immature core protein aa(1–191) remains in the cytoplasm and the processed, mature core protein is present in both the nuclear and cytoplasmic compartments, whereas the shorter core proteins aa(1–120) or aa(1–140) are targeted to the nucleus.

**Figure 2:**
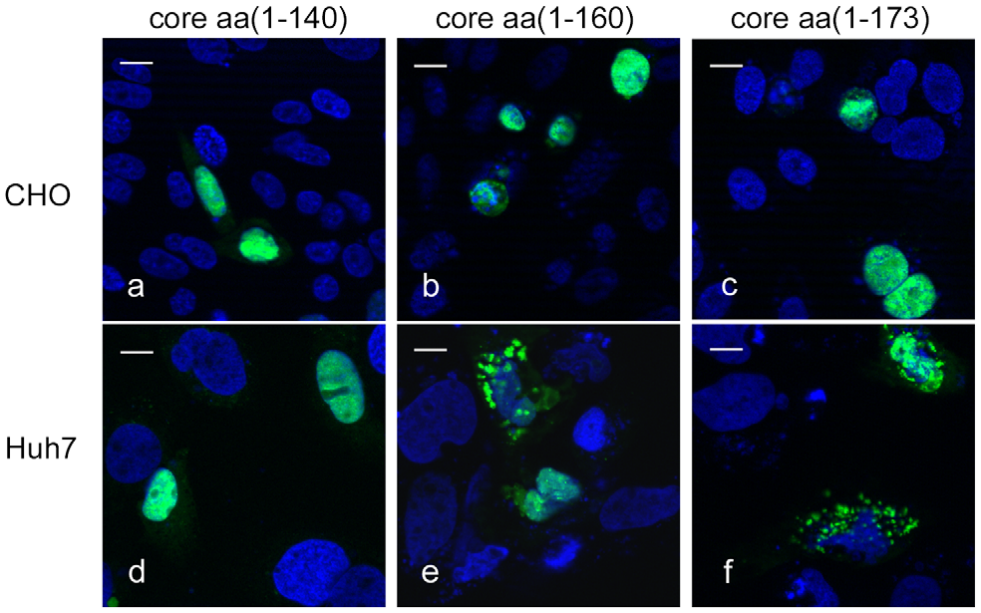
Subcellular localization of HCV core proteins of different lengths in CHO and Huh 7 cells. Plasmids encoding EGFP-labeled core proteins composed of aa(1–140) (a,d), aa(1–160) (b,c), and aa(1–173) (c,f) were used to investigate the subcellular distributions of the encoded proteins in the several human and non-human cell lines. Cells were transfected with plasmids encoding the corresponding EGFP-labeled proteins, grown for 24 h and analyzed by fluorescence microscopy, as described in the Materials and Methods section. (a–c) results obtained for CHO cell line (the same type of distribution was observed for ARL-6 and HEK-293 cells) and (d–f) distribution representative for Huh 7 cells (and other cells of hepatic origin as HepG2 or Fa2-N4). Bar represents 10 µm.

Using plasmids encoding EGFP-labeled core proteins composed of aa(1–173), aa(1–160) or aa(1–140), we investigated the subcellular distribution of the protein in various cell lines. The short form of core aa(1–140) was found in the nucleus in all cell lines tested. By contrast, core proteins aa(1–160) and aa (1–173) were found in both the cytoplasmic and nuclear compartments in transfected Huh 7 cells, but solely in the nucleus of cells of non human origin, such as Chinese hamster ovary cells (CHO) (Figure 2) and rat hepatoma cells (ARL-6) (not shown). Results identical to those for Huh7 cells were obtained for other hepatic cells (HepG2 hepatoma cell line and Fa2N4 immortalized human hepatocytes) in which HCV core proteins aa(1–160) and aa (1–173) were found in both the cytoplasmic and nuclear compartments. By contrast, in human non hepatic cells, such as embryonic kidney cells (HEK-293) these two core proteins were found exclusively in the nucleus (not shown), as for CHO and ARL-6 cells.

**Figure 3:**
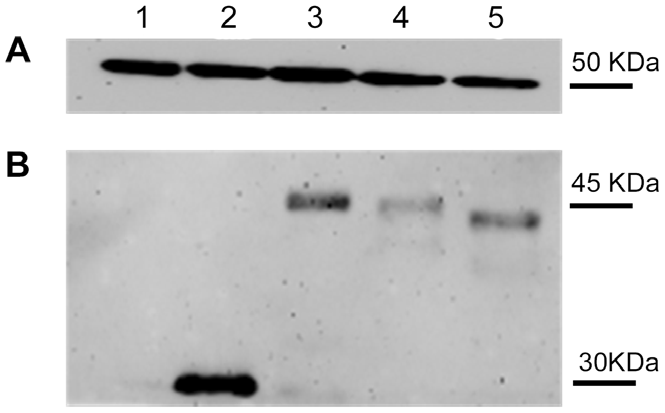
Western Blot analyses of constructs used for cell transfection. The integrity of the constructs used to transfect cells (shown in Figure 2) was assessed by Western Blotting. After separation of the proteins in cell lysates by SDS-PAGE, the bands were transferred to nitrocellulose membranes and for detection with anti-EGFP and anti-tubulin antibodies followed by peroxidase-labeled anti-rabbit IgG. The protein bands were detected by chemiluminescence. 1. Lysed Huh 7 cells; 2-Huh 7 cells transfected with pEGFPN1, 3-Huh7 cells transfected with core sequence aa(1-173) inserted into pEGFPN1; 4-Huh 7 cells transfected with the core aa(1–160) sequence in pEGFPN1; Huh 7 cells transfected with core sequence aa(1–140) inserted into pEGFPN1.

We performed Western Blot analyses of the EGFP-labeled core proteins generated, to check their integrity and to exclude the possibility of degradation of the constructs used for these experiments, which might affect the subcellular distribution of the corresponding proteins. These experiments (shown in Figure 3) confirmed that only one EGFP-labeled protein was produced in transfected Huh7 cells for each construct: a 47 kDa EGFP-labeled core protein for the plasmid encoding core aa(1–173), a 45 kDa EGFP-labeled protein for the plasmid encoding aa(1–160) and a 43 kDa EGFP-labeled protein for the plasmid encoding aa(1–140). There was therefore no degradation of the constructs and the distribution of core proteins was specific to the cells analyzed.

These results suggest that, in addition to the previously described NLS, there may be other signals in HCV core and interacting sequences in human hepatic cells, regulating the subcellular distribution of core protein. These mechanisms may be nonfunctional or differently regulated in non human and human cells of non hepatic origin.

### Identification of a “classical” NES in the immature form of HCV core

The subcellular distribution of the HCV core protein, like that of other viral proteins, may be influenced by the presence of nuclear localization signals (NLS) and nuclear export signals (NES). Indeed, the tripartite NLS located in the N-terminal DI have been described and shown to be functional. These and other functional domains of the core protein are shown in Figure 1. However, no NES capable of directing the translocation of the protein from the cell nucleus to the cytoplasm has ever been reported in HCV core.

**Figure 4:**
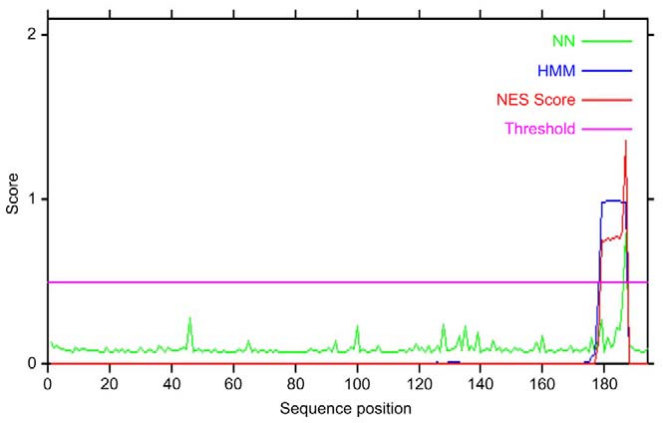
Identification of a classical NES in the immature form of HCV core protein. A leucine-rich NES with an LXL motif was identified in domain III of core, between aa179 and 187, with NetNES Software. This sequence is absent from the mature form of core, because it is cleaved by SPP during core processing. Graphical plot of the values (NES score) calculated by the prediction server from the Markov Model (HMM), and Artificial Neural Network (NN) scores. If the calculated NES score exceeds the threshold, then the residue concerned is predicted to be involved in a nuclear export signal.

We therefore used NetNES software (www.expasy.org) to search for a “classical” NES in the core protein. Such “classical sequences” are relatively short linear oligopetides enriched in leucine residues, usually consisting of the peptide sequence X-R(2-4)X-R2-X-R-X, where X is leucine, isoleucine or valine and R is any amino acid. This analysis identified a “classical” leucine-rich nuclear export sequence (NES Classic) within the C-terminal part of core, between aa 179 and 187 (Figure 4). Nevertheless, it is well established that sequential cleavages by signal peptidase and signal peptide peptidase remove the C-terminal hydrophobic DIII of core, to yield the mature form of core composed of aa(1–173), more recently defined as aa(1–177). Thus, the immature form core aa(1–191), attached to membranes, contains the classical NES, whereas the mature form of core does not (Figure 1).

### Identification of a “non classical” NES in HCV core

As the mature core (aa(1–173)) was found in both the cytoplasm and the nucleus, another “non classical” or “atypical” NES is probably present in the mature form of core. Such sequences have been reported for several viral proteins, such as equine infectious anemia virus (EIAV) and feline immunodeficiency virus (FIV) Rev.

**Figure 5:**
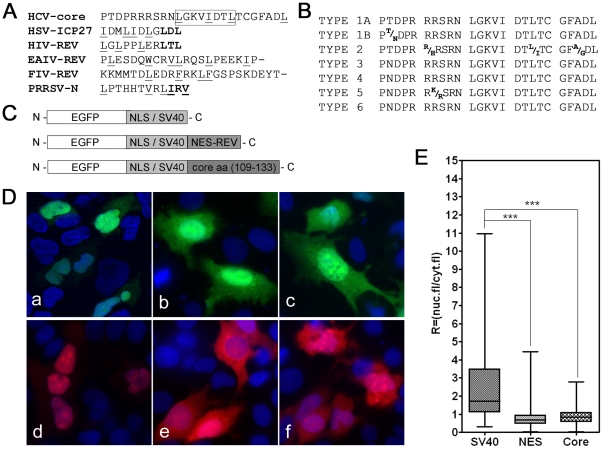
Identification of a functional, “non-classical” NES” in the core protein. (A) CLUSTAL W (1.81) software was used for multiple sequence alignment analysis, leading to the identification of a “non classical” NES sequence in domain II of the core protein. The potential NES signal aa(109–133) in core was compared with known viral NES sequences. Underlined regions correspond to hydrophobic amino-acid residues, and letters in bold typeface identify the conserved LXL motifs. The frame delineates a region of the export sequence containing amino-acid residues L(119), I(123) and L(126), which were replaced by alanine residues (the corresponding immunofluorescence analyses are shown in Figure 6). (B) The amino-acid sequences of the fragment corresponding to the putative NES aa(109–133) in HCV core proteins are well conserved in different HCV genotypes. The consensus sequences are shown, for each virus genotype, and were obtained by the alignment of 1245 sequences corresponding to the putative NES for HCV type 1A, 2078 sequences for HCV type 1B, and 95, 264, 60, 12 and 121 sequences for HCV types 2, 3, 4, 5 and 6, respectively. Sequences were obtained from the Los Alamos Data Bank (National Institutes of Health). (C) Schematic diagram of the plasmids used to investigate the functionality of the putative export sequence of core, aa(109–133). The SV40 NLS was used as a nuclear reporter, and the NES of the HIV Rev protein was used as a control export signal. The sequences shown were fused to either EGFP or m-Cherry, to allow the visualization of proteins in transfected cells. (D) Subcellular distribution of the proteins encoded by the plasmids depicted above. Huh7 cells grown on coverslips were transfected with the appropriate plasmids; 40 h after transfection, the cells were fixed in 4% PFA and examined by fluorescence microscopy. Panels a-c represent proteins labeled with EGFP, d-f the equivalent proteins labeled with m-Cherry. Proteins containing only the SV40 NLS were present mostly in the cell nuclei (a, d); proteins containing the control SV40 NLS and Rev NES were found in both the nucleus and the cytoplasm (b, e). The core sequence containing a putative NES, aa(109–133), is functional, because it was exported from the nucleus to the cytoplasm (c, f), like HIV Rev NES (b,e). Staining of nuclei with DAPI. (E) Graphical representation of nonparametric one-way ANOVA of the ratios of fluorescence between the nucleus and cytoplasm for the three plasmids. Cytoplasmic fluorescence is significantly higher for both EGFP-NLSSV40-NESRev and EGFP-NLSSV40-core aa(109–133).

We therefore searched for another, “non classical” NES in the mature form of core by generating multiple sequence alignments and comparing the core aa sequence with known viral sequences enriched in hydrophobic residues that can function as NES motifs, as described by Rowland *et al*. 2003 for the N–protein of porcine reproductive and respiratory syndrome virus (PPRSV). We found that the aa(109–133) region of core contained a sequence similar to that of other “atypical” viral NES. An analysis comparing the putative NES of core with other viral sequences is shown in Figure 5A.

**Figure 6:**
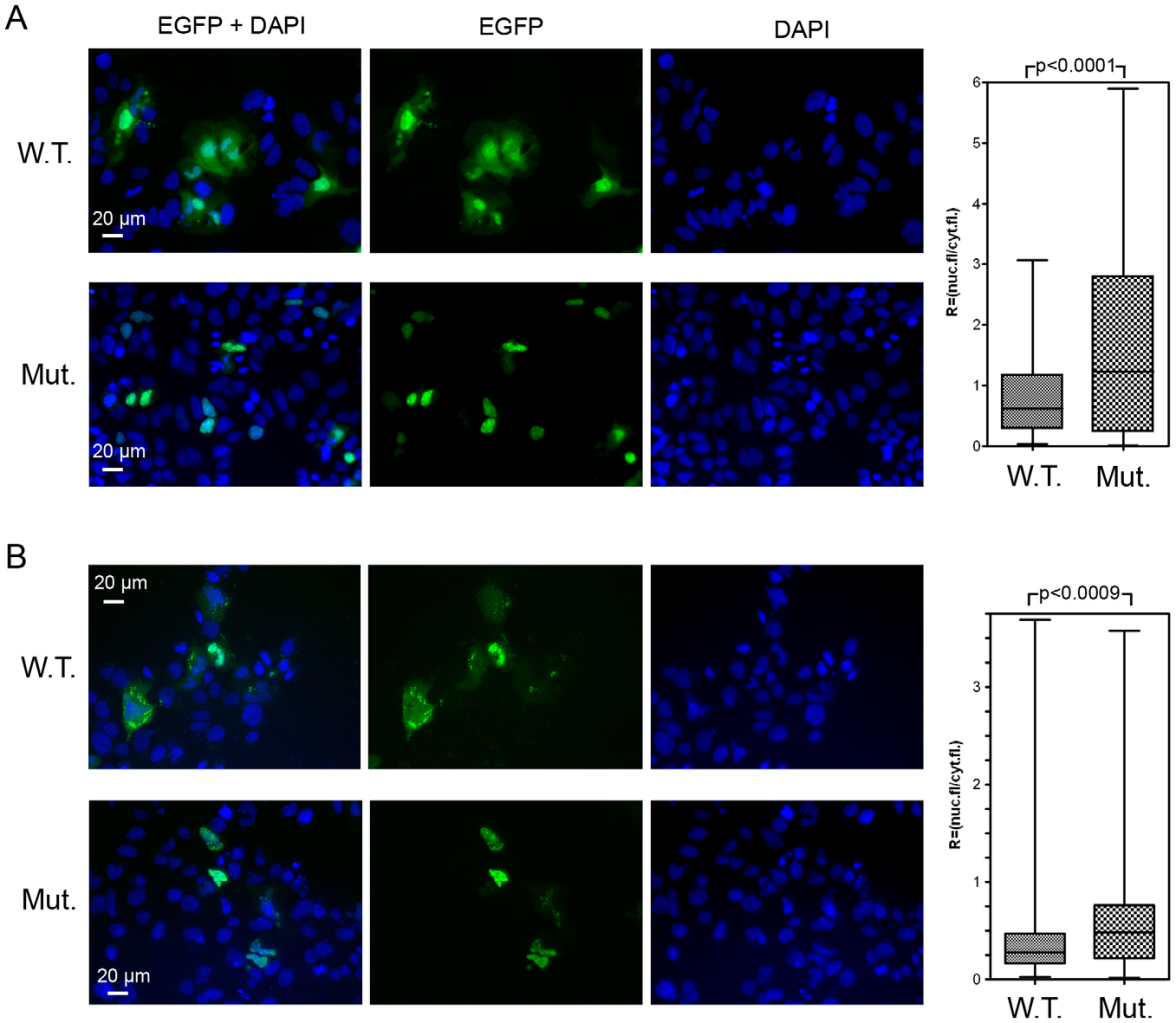
Fluorescence is predominantly nuclear for the protein encoded by the mutated NES. (A) The subcellular distribution of the proteins encoded by the construct with the mutated nuclear export sequence in HCV core was investigated by immunofluorescence. Mutations were introduced into the NES, as shown in Figure 5 A: amino-acid residues L(119), I(123) and L(126) were replaced by alanine residues (underlined within the frame). (A) Plasmids containing the EGFP, NLSSV40-core aa(109–133) and EGFP NLSSV40-mutated core aa(109–133) sequences were used to transfect Huh 7 cells, as described for Figure 5. After transfection (40 h), the cells were fixed with 4% PFA and examined by fluorescence microscopy. Upper panel represents immunofluorescence analyses of the subcellular distribution of the protein produced by the wild-type EGFP-NLSSV40-core aa(109–133) construct, lower panel shows the distribution of the corresponding construct with a mutated NES. The protein encoded by the wild-type construct (W.T) was found in both the nucleus and the cytoplasm; the protein encoded by the mutated construct (Mut) was found mostly in cell nuclei. Staining of the nuclei with DAPI. (B) EGFP-tagged core protein encoded by the wild type core aa(1–173) construct was found in both the nucleus and the cytoplasm (upper panel); the protein encoded by the mutated construct (Mut) was found mostly in cell nuclei (lower panel). Staining of the nuclei with DAPI. Graphical representation of subcellular distribution of the fluoresecence signal, based on a nonparametric *t*-test is shown on the right side of each panel. The graph shows the ratios of fluorescence between the nucleus and cytoplasm for the two plasmids encoding the wild-type and mutated proteins.

Comparative analyses of consensus sequences available for the core proteins of different genotypes provided evidence that this region was well conserved in various HCV genotypes (Figure 5B).

### The aa(109–133) sequence is a functional NES

We investigated whether the putative NES sequence (aa(109–133)) could direct the export of the protein from the nucleus to the cytoplasm, using an approach previously used to characterize the NES of PPRSV and HSV proteins. We constructed three plasmids (shown in Figure 5C). In the first, EGFP or mCherry was C-terminally tagged with the NLS of SV40 large T antigen, which targets proteins to the nucleus. The second plasmid encoded the SV40 NLS fused to the potential NES sequence of the HCV core protein aa(109–133). Finally, EGFP or mCherry fused to the SV40 NLS and the functional NES of the HIV Rev regulatory protein was used as a control for nuclear export. The Rev protein of HIV is a shuttling protein that promotes the nuclear export of mRNAs encoding viral structural proteins in a CRM-1-dependent manner.

We transiently transfected Huh7 cells with these plasmids and, after 40 h, we determined the subcellular distribution of the encoded proteins by fluorescence and confocal microscopy (Figure 5D). Fluorescent proteins containing the SV40 NLS were found almost exclusively in the nucleus, whereas proteins containing both the SV40 NLS and the Rev NES were found in both the nucleus and the cytoplasm. Similarly, the protein containing the SV40 NLS and the putative core NES was found in both the nucleus and the cytoplasm, providing evidence that aa(109–133) of core is a functional NES capable of directing the chimeric protein from the nucleus to the cytoplasm. Similar results were obtained with plasmids encoding proteins tagged either with EGFP or with mCherry, excluding the possibility that the subcellular distribution of the encoded proteins was dependent on the fluorescent tag used.

We confirmed the subcellular distribution of the putative NES of core (aa(109–133)) and of the proteins encoded by control plasmids, in quantitative analyses (Figure 5E). These analyses provided evidence that the aa(109–133) core sequence acted as a functional NES, targeting the protein from the nucleus to the cytoplasm, like the HIV Rev protein NES.

### Mutation of the NES sequence changes the subcellular distribution of HCV core

We hypothesized that the hydrophobic residues L(119), I(123) and L(126) in the core NES (Figure 5A, underlined within the frame) might be important for the nuclear export of this protein. We tested this hypothesis by carrying out comprehensive alanine-scanning mutagenesis and determining whether the replacement of these residues by alanine residues decreased the nuclear export of the protein, thereby resulting in accumulation of larger amounts of protein in the nucleus.

Cells were transfected with mutated constructs and the subcellular distributions of the resulting proteins were analyzed by immunofluorescence microscopy and compared with that of the non mutated control.

Transfection efficiency for the construct containing SV40 NLS fused to the mutated putative export sequence aa(109–133) of the core protein was similar to that for the wild-type construct, but the mutated protein had a different subcellular distribution. Indeed, whereas the wild-type chimeric protein with a putative export sequence aa(109–133) was found principally in the cytoplasm, the protein encoded by the mutated construct was mostly nuclear (Figure 6 A). Quantitative analyses confirmed that mutations affecting this core fragment significantly decreased the nuclear export of the protein produced and thus provided evidence that the aa(109–133) core sequence acted as a functional NES, targeting the protein from the nucleus to the cytoplasm.

### The C-terminal aa(140-160) sequence contributes to the cytoplasmic localization of core

We have demonstrated above that aa(109–133) is a functional NES. Consistent with this finding, core protein aa(1–120), which does not contain this sequence but does contain a functional NLS in the N-terminus of core, is found exclusively in the nucleus of hepatoma cells. However, another core fragment, aa(1–140), which includes the NES identified above, is also found exclusively in the nucleus, whereas larger core fragments, consisting of aa(1–160) or aa(1–173), were found in both the nucleus and the cytoplasm of hepatoma cells (Figure 2), as previously reported. We therefore conclude that, in addition to our NES, HCV core region aa(140–160) influences the subcellular distribution of the core protein.

The core protein sequence has been analyzed for determination of its secondary structure. Both aa(109–133) and aa(179–187) form α-helices. These sequences correspond to the two NES motifs identified in HCV core: the classical motif in DIII (Figure 4) and the “atypical” motif in core DII (Figure 5A). The adjacent aa(134–143) sequence was identified as a loop region, whereas aa(144–160) was also found to be rich in α-helices. This fragment may therefore influence the subcellular distribution of HCV core protein. It is thus possible that either there is another NES in this region that enhances the effect of the NES in aa(109–133) identified in this study, or that this sequence affects the subcellular distribution of core due to interaction with membranes and/or lipid droplets.

**Figure 7:**
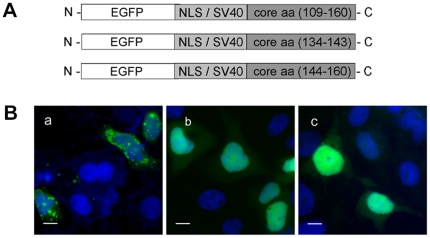
Core protein fragment aa(134–160) contains no other functional NES. (A) Three plasmids encoding the core fragments aa(109–160), aa(134–143) and aa(144–160) fused to EGFP were constructed as described in the Materials and Methods section. (B) Analysis of the subcellular distribution of the corresponding proteins encoded by the plasmids shown in (A). Cells were transfected with plasmids encoding corresponding EGFP-labeled proteins, grown for 24 h, fixed in 4% PFA and analyzed by fluorescence microcopy. Core protein fragment aa(109–160) was located in the cytoplasm (a), whereas proteins corresponding to core fragments aa(134–143) (b) and aa(144–160) (c) remained in the nucleus. Staining of the nuclei with DAPI. Bar represents 10 µm.

We investigated the possibility of another NES being present in the aa(109–160) region of core, by constructing three new plasmids encoding protein fragments aa(109–160), aa(134–143), and aa(144–160) of core (Figure 7A). Huh7 cells were transfected with these plasmids and, 40 hours later, we determined the subcellular distribution of the encoded proteins by fluorescence microscopy.

The aa(109–160) core protein fragment was found predominantly in the cytoplasm, whereas aa(134–143) and aa(144–160) were found solely in the nucleus (Figure 7B). These experiments showed clearly that aa sequences (144–160) and (134–143) did not function as additional NES. Indeed, in the absence of the aa(109–133) sequence identified in this study, these core protein fragments were not exported from the nucleus. We therefore conclude that the cytoplasmic distribution of core requires a functional NES within aa(109–133). However, the presence of this sequence is not sufficient and an additional fragment aa(140–160) is also required to maintain the protein in the cytoplasm.

The hydrophobic C-terminal DII of HCV core, containing this sequence, is required for the binding of core protein to lipid droplets and membranes, and for core protein folding and stability. Indeed, residues of two amphipathic helices, aa(119–136) and aa(148–164), in DII are critical for this association, as shown by extensive mutational analyses. Therefore, the core fragment aa(109–160) is found mostly in the cytoplasm, because, in addition to the NES, it mediates interactions of core with the cytoplasmic lipid droplets and/or membranes *via* specific sites in the aa(140–160) sequence.

### The nuclear export mediated by the core NES is CRM-1-dependent

Proteins containing NES are recognized by the CRM-1 export receptor, a member of the karyopherin superfamily of importin-β nuclear transport receptors. CRM-1 exports cargo proteins containing an NES from the nucleus to the cytoplasm. Leptomycin B is a potent and specific nuclear export inhibitor that alkylates CRM-1, specifically inhibiting the CRM-1-dependent nuclear export of proteins such as HIV Rev.

**Figure 8:**
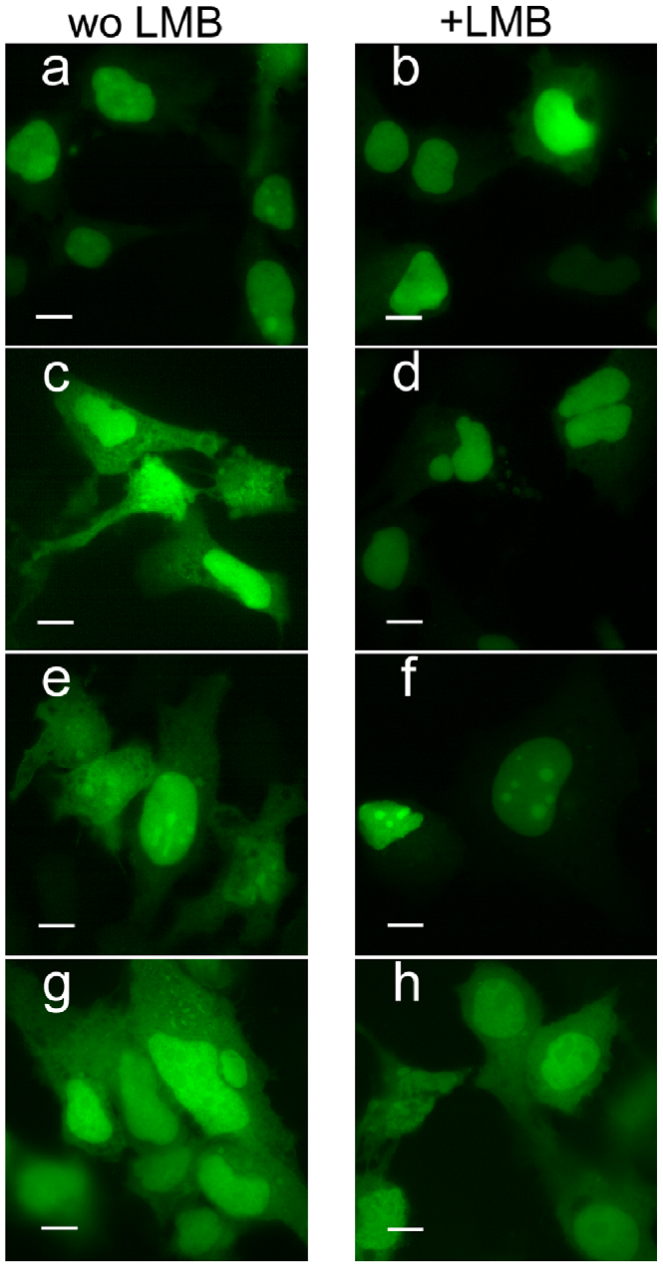
Nuclear export of core NES is mediated by CRM-1. Huh7 cells were transfected with a plasmid encoding EGFP-tagged NLS SV40 (a, b), EGFP-tagged NLS SV40 and HIV Rev NES (c, d) or the plasmid encoding EGFP-tagged NLS SV40 and putative core NES, aa(109-133) (e, f). Cells transfected with the EGFP-tagged control plasmid pEGFPC1 are shown (g, h). Twenty-two h after transfection, the cells were treated for 4 h with 10 ng/ml LMB (b, d, f, h; +LMB). The cells were subsequently washed, fixed and analyzed by immunofluorescence microscopy. The bar represents 10 µm.

We therefore investigated whether the nuclear export mediated by the NES in aa(109–133) was sensitive to LMB, and thus dependent on the CRM-1-dependent export pathway. Huh7 cells were transfected with a plasmid encoding this sequence and, 40 hours later, cells were treated with 2 or 10 ng/ml LMB. We found that, at both concentrations used, LMB inhibited the nuclear export mediated by the core aa(109–133) sequence (Figure 8). Nuclear export driven by the HIV Rev protein NES, used as a control, was inhibited by LMB to a similar extent, whereas aa sequences containing the SV40 NLS were always nuclear. The EGFP-tagged control protein was found in both the cytoplasm and nucleus, and this passive distribution was not sensitive to LMB treatment. The aa(109–133) region of the core protein may therefore be considered a functional NES, operating in a CRM-1-dependent manner.

**Figure 9:**
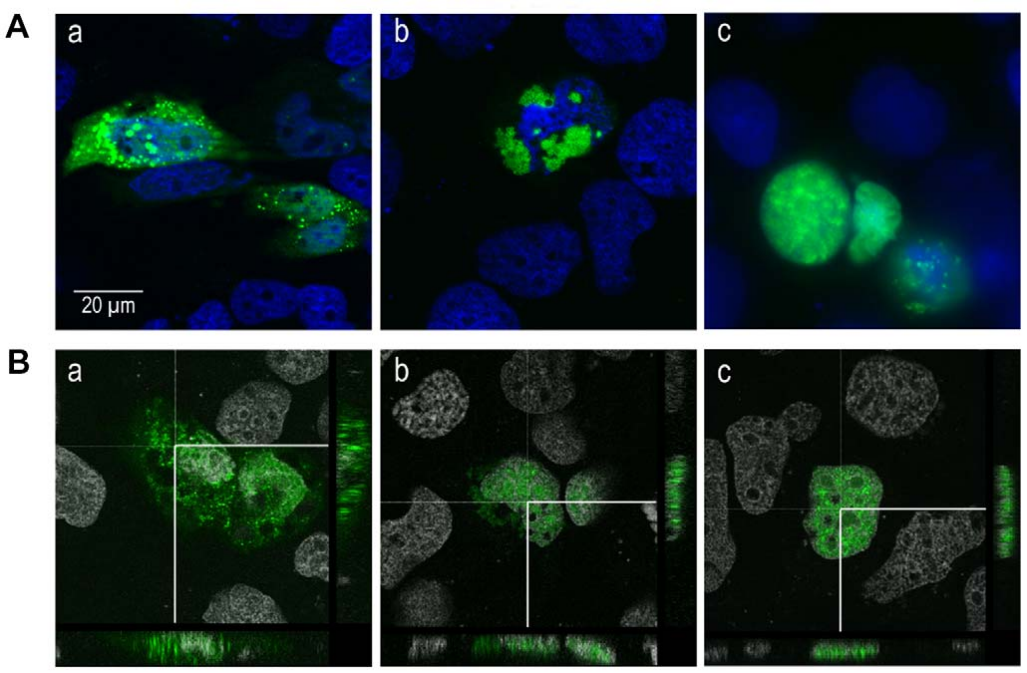
Subcellular localization of core in LMB-treated cells. (A) Production of the EGFP-labeled core protein aa(1–173) in Huh7 cells results in the presence of this protein in both the cytoplasmic (a) and nuclear (b) compartments. Huh7 cells were transfected with a plasmid encoding EGFP–labeled aa(1–173) protein and, 40 h later, cells were treated with 10 ng/ml LMB. The inhibition of nuclear export by LMB induced an increase in the accumulation of HCV core protein in the nucleus (c). (B) Subcellular localization of core protein, with and without LMB treatement, confirmed by confocal microscopy analyses. Huh7 cells were transfected with a plasmid encoding the EGFP-labeled core protein aa(1–173), as described in (A). Slides correspond to the panels shown in A: the cytoplasmic (a) and nuclear (b) distribution of the core protein in non treated cells and its nuclear localization after LMB treatment (c). The bar indicates 20 µm.

Following the transfection of Huh7 cells with a plasmid encoding an EGFP-labeled core aa(1–173), the protein was found in both the nucleus and cytoplasm, unless translocation had been blocked by LMB. This treatment increased the proportion of core found in the nucleus although the protein was also present in the cytoplasm in several cells (data not shown). The subcellular distributions of core protein in the presence and absence of LMB were confirmed by confocal microscopy (Figure 9).

In addition, mutation of the hydrophobic residues L(119), I(123) and L(126) in the NES region of core protein aa(1–173) significantly increased the nuclear localization of this protein, to levels greater than observed for the wild-type protein (Figure 6B).

Thus, the mature core protein produced in transfected hepatoma cells is located in both the cytoplasmic and nuclear compartments. The translocation of the protein from the nucleus follows the CRM-1-dependent pathway, because it is inhibited by LMB. Similarly, mutations of the three hydrophobic amino acids of the NES region spanning aa(109–133) significantly increase the nuclear localization of the core protein.

### Detection of core in the nucleus by electron microscopy

**Figure 10:**
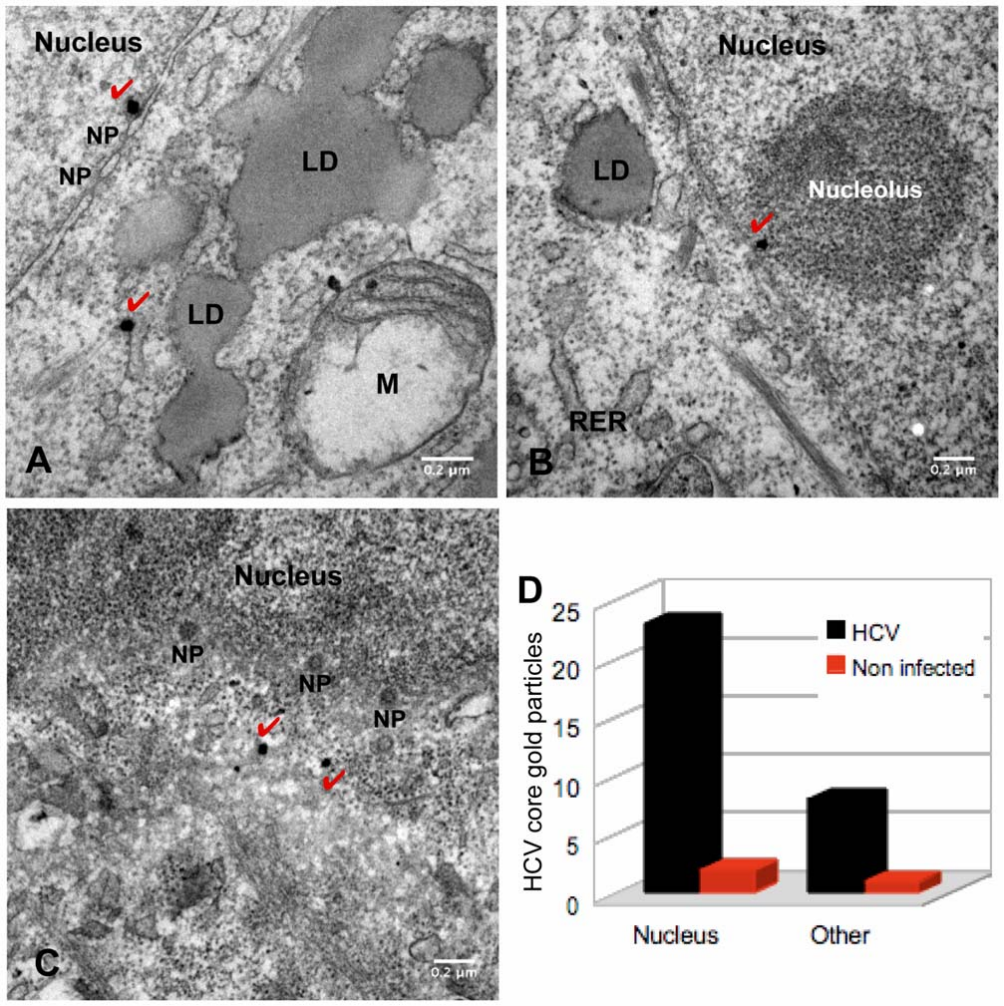
Nuclear localization of core protein in HCV infection, as demonstrated by electron microscopy. For electron microscopy analysis, virus particles produced in Huh 7.5 cells were concentrated from the cell supernatant by centrifugation through a sucrose cushion for 4 h at 32,000 rpm in an SW 32 Ti rotor. Concentrated virus preparation was incubated with Huh 7.5 cells at 4°C. The cells were then transferred to 37°C and incubated for a further 20 min. Cells were washed, fixed with 4% PFA and stained with monoclonal anti-core antibody ACAP27, followed by secondary, colloidal gold-labeled anti-mouse IgG. Nanogold staining was enhanced by incubation with the HQ silver enhancement kit, and cells were post-fixed by incubation with 1% osmium tetroxide (for details, see the Materials and Methods section). Non infected cells were also incubated with anti-core antibodies. Panels A, B and C, show representative pictures of HCV core localization in HCVcc-infected cells. The presence of one silver-enhanced gold particle is indicated. LD, lipid droplet; M, mitochondrion; NP nuclear pore. (D) Quantitative evaluation of HCV core labeling (gold particles) in the perinuclear *vs* other areas of HCV-infected and non infected cells, from randomly selected intracellular zones of equivalent area. Thirty cells were considered for each analysis.

In a recent study of HCV infection, we detected HCV particles by immunoelectron microscopy during the entry of the virus into Huh 7.5 cells (*P.Maillard, M.Walic et al. PloS One in press*). In addition, core and some non structural proteins of other Flaviviruses are trafficked to the nucleus at very early phase of infection. These findings prompted us to investigate whether HCV core protein could also be directed to the nucleus at such early stages of infection. For electron microscopy analysis, virus particles produced in Huh 7.5 cells were concentrated by pelleting through the sucrose cushion and cells were infected with concentrated virus preparations as described in the Materials and Methods section. Virus core protein was stained with the ACAP27 monoclonal antibody, followed by an anti-mouse IgG secondary antibody labeled with colloidal gold. HCV core protein was observed in the cell nucleus 20 minutes after infection, where it was located close to the nuclear membrane or the nuclear pores, as shown by immunostaining with anti-core antibodies (Figure 10 A–C). Taking into account the time after infection, the intranuclear core protein was almost certainly derived from internalized virus particles rather than represented HCV core protein produced de novo in infected cells.

Quantitative analyses performed on HCV-infected and non infected cells after staining with anti-core antibodies confirmed the specificity of our observations and showed that a larger proportion of the gold-labeled HCV core was present in the cell nucleus than elsewhere in the cell at this stage of infection (Figure 10 D). No such staining was observed when unrelated monoclonal antibodies were used. These observations suggest that, at very early stages of HCV infection, at least some of the HCV core protein is directed to the nucleus.

### Detection of core in the nucleus by immunofluorescence analysis after LMB treatment

In most studies in the HCVcc in vitro replication system, HCV core protein has not been found in the nucleus. However, if PA28γ, an activator of the proteasome, which is responsible for ubiquitin-independent degradation in the nucleus, was inhibited or absent, very small amounts of core could be detected, by immunofluorescence microscopy, in the nuclei of HCV-infected Huh7 cells.

We investigated whether the NES identified was functional in the JFH1 HCVcc cell culture model. Immunofluorescence microscopy detected HCV core in the cytoplasm of infected cells, but no evidence of a nuclear localization of core was obtained at various times after infection, consistent with previous findings. We therefore used a small interfering RNA to silence the PA28γ gene, to determine whether this treatment increased the amount of core protein in the nucleus, as previously reported.

**Figure 11:**
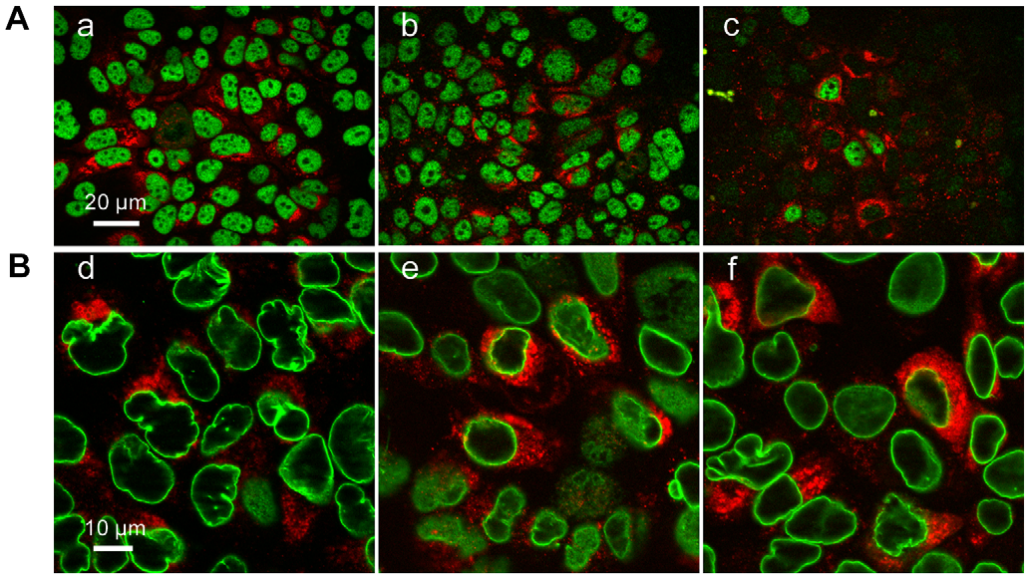
Cytoplasmic localization of core in HCV-infected cells by immunofluorescence without LMB treatment. (A) *Silencing of the proteasome activator PA28γ*. Huh 7.5 cells were transfected with an siRNA targeting PA28γ or a control non targeting siRNA 18 h before infection with JFH1. For analysis of the expression of PA28γ by immunofluorescence, cells were stained with rabbit anti-PA28γ antibody, followed by Alexa Fluor 488-conjugated anti-rabbit IgG. Staining for HCV core was carried out 48 h after infection, with the monoclonal anti-core antibody ACAP-27, followed by Alexa Fluor 568-tagged anti-mouse IgG (in red). (a) Non treated HCV (JFH1)-infected Huh 7.5 cells; (b) HCV-infected cells transfected with control, non-targeting siRNA before infection; (c) cells with PA28γ knockdown due to transfection with a specific PA28γ-targeting siRNA. (B) Expression of core in Huh7.5 cells after silencing of the PA28γ proteasome activator. JFH1-infected cells were stained with rabbit anti-lamin B antibody and Alexa Fluor 488-conjugated anti-rabbit IgG as a secondary antibody, to outline the cell nuclei, and with ACAP27 anti-core antibody followed by Alexa Fluor 568-conjugated anti-mouse IgG, for subcellular localization of HCV core. (d) JFH1-infected Huh 7.5 cells without PA28γ silencing, (corresponding to the image shown in (a) panel A); (e) HCV-infected cells transfected with control, non-targeting siRNA before infection (corresponding to the image shown in (b) panel A); and (f) cells with PA28γ knockdown with a PA28γ-specific siRNA before infection with HCV (corresponding to (c) in panel A). Staining of the nuclear membrane with anti-lamin B (green) and with anti-core antibody (red), as described above. No nuclear staining of core was detected, in either siRNA-silenced cells or in cells transfected with a control si-RNA.

PA28γ expression was successfully transiently knocked down by a specific siRNA targeting PA28γ, but not by a control, unrelated siRNA (Figure 11A). Despite careful analysis, no core was detected, by immunofluorescence microscopy, in the nucleus of either siRNA-silenced cells or cells transfected with a control RNA (Figure 11B). Instead, HCV core protein was concentrated in the perinuclear area of the cell, in both silenced and untreated cells.

**Figure 12:**
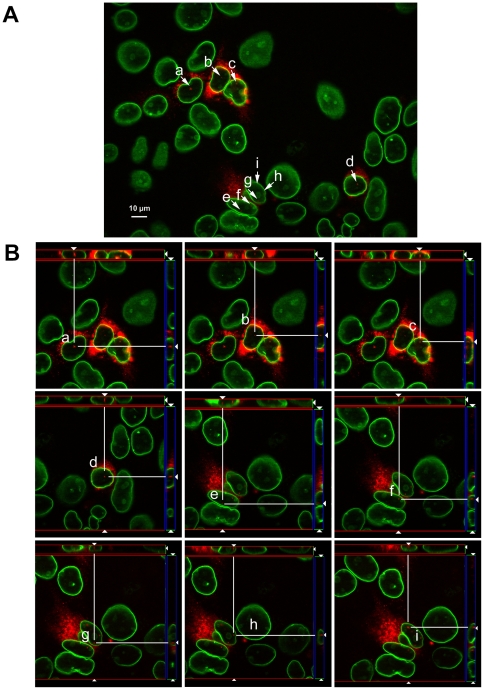
Intranuclear localization of core in HCV-infected cells treated with LMB. Huh 7.5 cells were infected with the JFH1 strain of HCVcc and, 2 h after infection, 10 ng/ml LMB was added, with maintenance of this concentration of LMB in the cell culture until 48 h post infection. (A) Detection of intranuclear core in LMB-treated cells. Several cells are shown in which core is present in the nucleus (designated a-i). These cells were analyzed further: 18 cross sections of images were taken to visualize the intranuclear localization of core, at different levels within the nucleus. (B) Cut view of Z stacks from the cells shown in A with the same designation (a–i). Images were captured with an Axioplan 2 microscope with Apotome (Zeiss) and Axiovision 4.6. Orthogonal views were obtained from Z-stack images, with a resolution of 0.33 µm. The nuclear membrane is stained with rabbit anti-lamin B antibody, followed by anti-rabbit IgG tagged with Alexa Fluor 488 (in green), for better visualization of the intranuclear localization of core. HCV core protein is stained with anti-core ACAP27 antibody, followed by Alexa-Fluor 568 labeled anti-mouse IgG (in red). Bar represents 10 µm. Negative controls for these studies (without LMB treatment) are shown in Figure 11, in which no core was detected in the nucleus, whether or not PA28γ was silenced.

Nevertheless, the treatment of infected cells with LMB, which was maintained in the culture medium for several hours to block the nuclear export of core protein, resulted in the detection of core within the nucleus in several cells, distributed as single points at several sites in the nucleus (Figure 12). We detected HCV core in the nucleus only when LMB was added at early time points (2–4 h) after infection, but not when the drug was added 48 hours after infection.

These findings clearly demonstrate that at least some core protein is targeted to the nucleus in the JFH1 HCVcc infection model, at early stages of infection. Core seems to be exported from the nucleus via the CRM-1-dependent pathway, because a nuclear distribution of this protein was observed only in LMB-treated cells.

### Leptomycin B affects HCV production

**Figure 13:**
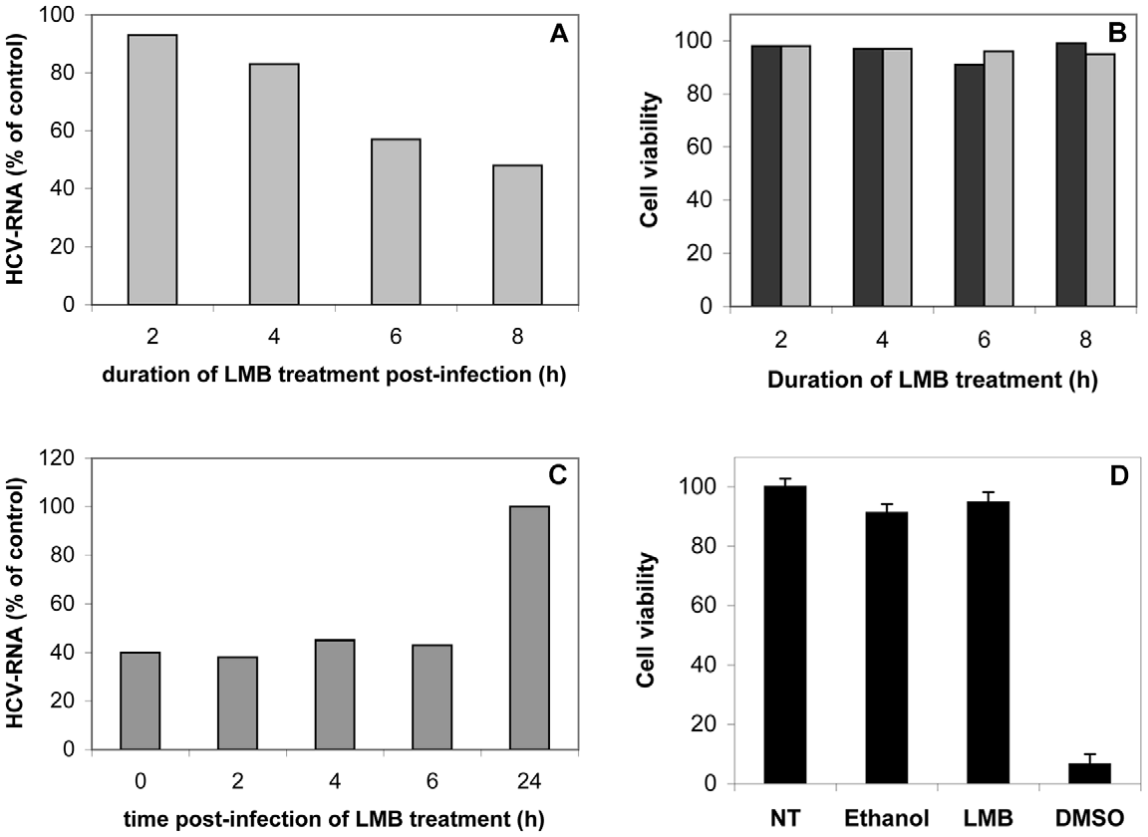
LMB treatment influences virus production. (A). Cells were treated with 10 ng/ml LMB for various time periods to determine the incubation time required to affect infection. Cells were infected with HCVcc (JFH1) and the drug was added immediately after infection. Cells were grown in the medium containing LMB, for the time indicated and HCV RNA was then extracted and quantified by RT-qPCR. The values are expressed as a percent of the amount of HCV RNA present in cells grown without LMB. (B). Control experiments carried out to demonstrate that the incubation of cells for 2–8 h with 10 ng/ml LMB (shown in (A) had no toxic effect on cell viability. Cell viability after LMB treatments was determined by counting live and dead cells after trypan blue staining, or by measuring cellular ATP present in culture wells as described in Materials and Methods. Untreated cells and cells treated with 10% DMSO (to induce cell death) were used as negative and positive controls, respectively. The results are expressed as a percent of the value obtained for an untreated control. (C). Treatment with LMB early in infection significantly decreases infection levels. Cells were infected with HCVcc 2 h at 37°C in the presence of 10 ng/ml LMB (T0) or infected with HCVcc and then treated with the drug at the indicated time points after infection (2 h, 4 h, or 6 h) and then incubated for a further 8 h. The treatment of cells with LMB 0–6 h post infection significantly decreases infection levels, whereas the same treatment (for 8 h) applied 24 h after infection has no effect on intracellular HCV RNA levels, as shown by comparison with the untreated control. (D) Control experiments for (C) showing that the application of LMB or its solvent (ethanol) at the same concentrations and for the same time period as used for (C) does not influence cell viability, as demonstrated by comparison with an untreated control. DMSO (at a concentration of 10%) was used as a positive control, to decrease cell viability. Values are expressed as a percent of untreated control.

As core was detected in the nucleus of HCV-infected cells treated with LMB, we investigated the possible effects of LMB treatment on virus multiplication. We infected Huh 7.5 cells with HCVcc in the presence or absence of 10 ng/ml LMB or added the drug to the cell supernatant at various time points after infection. Cells were then grown in the medium containing LMB until 32 hours post infection, and intracellular HCV RNA was quantified by RT-qPCR. Incubation with the drug for eight hours was needed for efficient inhibition of infection (Figure 13 A). Infection levels were about 60% lower than control infection levels when the drug was added during the first six hours of infection. By contrast, the percentage inhibition was only 20% if LMB was added eight hours after infection (not shown) and 0% if added twenty-four hours after infection (Figure 11 C). Control experiments excluded a cytotoxic effect of LMB or its solvent on Huh 7.5 cells (Figure 13 B, D).

Overall, these data showed that early LMB treatment influenced HCV RNA production, suggesting that the early shuttling of core between the cytoplasm and the nucleus might be important for virus multiplication.

## Discussion

We provide here the first demonstration that a functional NES is present in HCV core protein. We show that amino acids 109 to 133 are responsible for the active export of the HCV core protein out of the nucleus, *via* a CRM-1–mediated nuclear export pathway. This NES was functional in transfected cells and in an *in vitro* model of HCV replication (HCVcc). The trafficking of core protein into the nucleus early in infection may help to establish infection and facilitate the interaction of core with nuclear molecules, with potentially important pathological consequences.

HCV core protein is thought to be a major viral factor promoting liver disease during HCV infection and the malignant transformation of hepatocytes, leading to the development of HCC through interactions with several host cell factors involved in a wide range of cellular processes. Indeed, in various experimental systems, HCV core has been reported to affect transcription mediated by various gene promoters and apoptosis, thereby contributing to cell transformation. The oncogenic activity of core might be related to its nuclear localization. In liver biopsy samples from HCV-infected patients, HCV core has been found mostly in the cytoplasm, being only rarely detected in the nucleus of infected hepatocytes. Nevertheless, a nuclear location of truncated core proteins was detected in tumor tissues from patients with HCV-related hepatocarcinoma. Similarly, the nuclear accumulation of core has been observed in transgenic mice producing the HCV core protein and developing HCC.

Taking into account the role of HCV core as a viral factor of major pathological significance and understanding the mechanisms regulating its subcellular distribution and trafficking are of critical importance. Several studies on transfected cells have shown the HCV core protein to be located in the cytoplasm or nucleus, depending on its length. Consistent with this dual localization, many studies have reported interactions with molecules located in either the cytoplasm or the nucleus.

Our findings confirmed that, in an *in vitro* transfection system based on human Huh7 and HepG2 hepatoma cell lines or immortalized Fa2-N4 human hepatocytes, the aa(1–173) and aa(1–160) core proteins were found in both the nucleus and the cytoplasm. These proteins were found exclusively in the nuclei of non human cells (CHO, ARL-6) and in human cells of non hepatic origin (HEK-293). By contrast, the shorter core proteins aa(1–120) and aa(1–140) were found exclusively in the nucleus. Thus, the nuclear/cytoplasmic subcellular distribution of core proteins aa(1–173) and aa(1–160) was specific to human cells of hepatic origin. Our observations suggest that core protein may contain signals for specific transport mechanisms controlling its distribution between the nucleus and the cytoplasm that are functional in human hepatic cells.

The differences in the distribution of the protein between the nucleus and cytoplasm in the cell types tested may reflect the availability and/or functionality of the carrier proteins in these cells. Indeed, the subcellular distribution of a given protein may be controlled by the differential expression of carrier proteins in various tissues or host species, and may depend on the differentiation status of the cell.

The nucleocytoplasmic trafficking of various proteins and RNA is controlled by importins and exportins (also called karyopherins) from the importin-β superfamily of proteins. These proteins can therefore gain entry into the nucleus only if they possess the appropriate NLS recognized by nuclear importin receptors, or if they react directly with the nuclear pore complex. Consistent with the nuclear localization of core in several experimental systems, three NLS have been identified in the N-terminal domain of this protein. These signals consist of clusters of basic amino acids in the aa(5–13), aa(38–43), and aa(58–71) regions; they are functional and able to target core to the nucleus.

For reentry into the cytoplasm, proteins must contain the sequences required for interaction with export factors (exportins), enabling them to leave the nucleus *via* the nuclear pore. The nuclear export of proteins is mediated mostly by NES, leucine-rich aa sequences recognized by soluble export receptors, such as Exportin1 (CRM-1). No functional export signal, directing translocation of the core protein from the nucleus to the cytoplasm has been identified in the core sequence to date, although the presence of such a signal has been suggested.

We used NetNES software to search for a classical leucine-rich NES in HCV core, and found such a signal in the C-terminal part of the immature form of core at aa(179–187). This protein is always found in cytoplasmic compartment and, thus, the role of this signal remains unclear. In addition, this NES sequence is removed when the mature core protein is generated by cellular peptide peptidase processing.

In addition to this classical leucine-rich NES in the C-terminus of the immature core protein, we identified a second, “atypical” NES in the mature form of core, by sequence alignment, as previously reported by Rowland *et al*. for the identification of NES in the PPRSV nucleocapsid protein. Indeed, multiple sequence alignments of the HCV core with other viral NES, generated with CLUSTAL W, led to the identification of a candidate NES sequence at aa(109–133). Consistent with the putative biological role of this NES, this sequence contains a cluster of hydrophobic residues with a sequence similar to those of the NES of HSV ICP27, HIV Rev, equine infectious anemia virus Rev, feline immunodeficiency virus Rev and porcine reproductive and respiratory syndrome virus N protein. Moreover, this signal was well conserved in consensus sequences from various HCV genotypes.

We provide evidence that the NES identified at aa(109–133) is functional in transfected hepatoma cells and can export core protein fragments from the nucleus to the cytoplasm. First, a peptide corresponding to aa(109–133) of HCV core, like the NES of the HIV Rev protein, counteracted the nuclear translocation driven by the strong NLS of SV40 large T-antigen. The nuclear export of this NES, observed by fluorescence microscopy, was confirmed by quantitative analyses. LMB treatment inhibited nuclear export of the core NES, demonstrating that the aa(109–133) nuclear export signal functions in a CRM-1-dependent manner. Indeed, “atypical” NES sequences may also function in a CRM1-dependent manner.

Export signals essentially consist of several closely spaced leucine residues, but other hydrophobic amino acids, such as methionine, isoleucine, valine, phenylalanine and tryptophan, may replace leucine in the recognition motifs. Using comprehensive alanine-scanning mutagenesis we showed that the replacement of the Leu(119), Ile(123) and Leu(126) residues by alanine modified the export capacity of our NES. Indeed, whereas the wild-type chimeric protein encoded by the plasmid (EGFP-NLSSV40-NEScore) carrying the intact aa(109–133) sequence was exported into the cytoplasm, the protein encoded by the mutated construct was mostly found in the nucleus. These observations, validated by quantitative analyses, confirmed that the aa(109–133) core sequence acted as a functional NES, targeting the protein from the nucleus to the cytoplasm.

The functionality of the NES at aa(109–133) was further analyzed in the context of mature HCV core protein. Firstly, LMB treatment also modified the subcellular distribution of core aa(1–173), increasing its accumulation in the nucleus. Secondly, mutations of the hydrophobic residues Leu(119), Ile(123) and Leu(126), with the replacement of these residues by alanine residues, modified the export capacity of the protein. Our quantitative studies confirmed that the proportions of the protein located in the nucleus (as opposed to the cytoplasm) were significantly higher for the mutated protein than for the wild-type protein. These data provide experimental evidence for a role of the identified NES in the CRM-1-dependent nuclear export of HCV core.

CRM-1 has been identified as the export receptor, the principal mediator of nuclear export, allowing the nuclear-cytoplasmic shuttling of proteins and RNA between cellular compartments. CRM-1-dependent transport is well conserved throughout eukaryotes and LMB is a recognized inhibitor of the active export of most molecules from the nucleus. Indeed, the “steady-state” localization of proteins does not always reflect the biological importance of their site of action. The use of LMB to block CRM-1-mediated nuclear export results in the detection of the NES-containing protein in the nuclear compartment, although its “steady-state” location appears to be exclusively or predominantly cytoplasmic, with the equilibrium of bidirectional transport favoring nuclear export. Nevertheless, CRM-1-mediated transport is a highly regulated process and this regulation includes the masking of NES, phosphorylation and heterodimerisation of the protein and the formation of disulfide bonds by an oxidative process. The availability of specific cofactors may also influence this regulation. The presence of such cofactors may contribute to the observed differences in the subcellular distribution of core proteins in various cell types (see above).

In our study, both the NES aa(109–133) and the adjacent hydrophobic sequence in domain II were required for a cytoplasmic distribution of core. Indeed, we found that only a relatively long core protein fragment, aa(109–160), was located in the cytoplasm, with shorter core fragments lacking either NES or this adjacent hydrophobic sequence being found in the nucleus. We showed that no other NES capable of enhancing core export from the nucleus was present in the adjacent (133–160)aa sequence. However this core fragment, in addition to the NES, was necessary for a cytoplasmic distribution of the protein. As this hydrophobic core fragment contains specific sites interacting with LDs and membranes, these interactions are probably required, in addition to our NES aa(109–133), for the maintenance of core protein in the cytoplasm. Consistent with this notion, the aa(120–150) core protein (containing a hydrophobic fragment interacting with LDs/membranes but not the NES) was found in the nucleus rather than the ER. Similarly, C-terminally truncated core proteins with NES, but lacking the hydrophobic fragment of DII, accumulated in the nucleus, thereby probably contributing to the development of HCC. Collectively, our observations indicate that both a nuclear export signal aa(109–133) and the adjacent hydrophobic sequence in domain II are required to target core protein to the cytoplasm and to keep it in this compartment.

The identification of a NES that was functional in transfected Huh7 hepatoma cells raised questions about the role of this signal in HCV infection. In the context of the infectious cell culture model in Huh7.5 cells (HCVcc), HCV core protein was found to colocalize with lipid droplets and membranes, but was not detected in the nucleus in most studies. However, knockdown of the PA28γ proteasome activator gene, blocking ubiquitin-independent nuclear degradation, led to the detection of a very small quantity of HCV core in the nuclei of infected Huh7 cells on immunofluorescence analysis.

Our key findings for transfected cells are consistent with the data obtained in the HCVcc replication model. First, our immunoelectron microscopy studies based on staining with anti-core antibodies provided evidence for a nuclear location of core as early as 20 minutes after the start of infection. This suggests that the nuclear trafficking of core takes place very early in the viral cycle, shortly after internalization of the virus. Second, the use of LMB to block CRM-1-dependent export resulted in the detection of HCV core protein in the nuclei of a number of JFH1-infected cells on confocal microscopy. Nuclear staining for HCV core was observed as several “spots” when various nuclear images (virtual cross sections) were analyzed by confocal microscopy. In particular, core was detected within the nucleus only if LMB treatment was applied early in infection (2 h post infection), and no core was detected in the nucleus if LMB was applied late in infection (48 h post infection). Intranuclear core was also observed in Huh7.5 cells in which PA28γ expression was knocked down but only after LMB treatment.

Early LMB treatment also decreased HCV RNA production, suggesting that the early shuttling of core between the cytoplasm and the nucleus may be important for virus multiplication. LMB had no toxic effect on the cells tested, but we cannot rule out the possibility that LMB treatment also influences other cell processes, in addition to core protein shuttling.

We conclude that the NES identified in HCV core protein is functional in the HCVcc replication system. Our electron microscopy studies suggest that some HCV core, derived from invading virus particles, is transported into the nucleus at very early stages of the viral life cycle. In addition, a fraction of core protein can be detected by immunofluorescence, in the nucleus, a few hours after infection, subsequently being exported to the cytoplasm in a CRM-1-dependent manner, as this export is blocked by LMB, a drug widely used to dissect nuclear export pathways.

Examples of viral proteins known to shuttle through the nuclear pore complex and for which the CRM-1-dependent pathway is known to export the corresponding viral RNA include HIV Rev and T-cell leukemia virus type 1 Rex. Structural and nonstructural proteins of several members of the flavivirus family, such as Japanese encephalitis virus (JEV), Dengue virus (DENV), and Kunjin virus (KUN), have also been shown to be actively translocated to the nucleus or to the nucleoli of infected cells, even when these viruses multiply entirely in the cell cytoplasm. This phenomenon may affect virus infectivity or disease pathogenesis. Indeed, DENV NS5 RNA polymerase can be detected in the nucleus very shortly after infection, and this protein is exported from the nucleus in a CRM-1-dependent manner. Nuclear NS5 suppresses the production of IL-8, a cytokine playing an important role in the antiviral response.

DENV core protein also localizes to the nucleus (and nucleoli) at very early stages in the viral life cycle, due to its bipartite NLS. The mechanisms of DENV nuclear export remain unknown, as this process is insensitive to LMB, suggesting that it does not require a functional NES [65]. Indeed, many other pathways exist for protein import and export, including the calreticulin pathway. Nevertheless, the nuclear localization of DENV core may regulate its replication cycle and apoptosis (see for review. Consequently, the construction of recombinant vaccines based on viral proteins deficient in nuclear trafficking signals could potentially lead to attenuation of the virus.

Our findings are consistent with the hypothesis that at least some HCV core protein is trafficked between the cytoplasmic and nuclear compartments early in HCV infection. Recombinant viruses mutated in the NES region investigated here, showed impaired virus production, producing less than 1% of the wild type virus in the HCVcc *in vitro* model. These observations provided evidence that this sequence is of importance for virus life cycle. The absence of the NES identified in this study and/or of the hydrophobic fragment of domain II (which act together to keep the protein in the cytoplasm) may account for the nuclear localization of the C-terminally truncated core proteins in patients with HCV-induced HCC and contribute to the cell transformation.
